# Sexual Dimorphism and Foraging Trips of the Laysan Albatross (*Phoebastria immutabilis*) on Guadalupe Island

**DOI:** 10.3390/ani9060364

**Published:** 2019-06-17

**Authors:** Julio César Hernández Montoya, Maricela Juárez-Rodríguez, Federico Méndez-Sánchez, Alfonso Aguirre-Muñoz, Evaristo Rojas-Mayoral, Eduardo Íñigo-Elias, Patricia Galina-Tessaro, Gustavo Arnaud, Alfredo Ortega-Rubio

**Affiliations:** 1 Centro de Investigaciones Biológicas del Noroeste (CIBNOR), La Paz 23090, Baja California Sur, Mexico; pgalina04@cibnor.mx (P.G.-T.); garnaud04@cibnor.mx (G.A.); aortega@cibnor.mx (A.O.-R.); 2 Grupo de Ecología y Conservación de Islas (GECI), Ensenada 22800, Baja California, Mexico; maricela.juarez@islas.org.mx (M.J.-R.); federico.mendez@islas.org.mx (F.M.-S.); alfonso.aguirre@islas.org.mx (A.A.-M.); evaristo.rojas@islas.org.mx (E.R.-M.); 3 Cornell Laboratory of Ornithology, Ithaca, NY 14850, USA; eei2@cornell.edu

**Keywords:** Laysan albatross, Guadalupe Island, sexual dimorphism, foraging trips

## Abstract

**Simple Summary:**

We evaluated the existence of sexual dimorphism in Laysan albatross from Guadalupe Island. Males were larger than females across all the morphological variables analyzed. We created a sex predictor model for Laysan albatross individuals that requires a minimum number of input variables and will considerably reduce the handling times and field costs of future studies. Laysan albatross foraging trips were recorded during their breeding season over multiple years and no significant differences were found between the distances travelled by males versus females.

**Abstract:**

Sexual dimorphism in the Laysan albatross (*Phoebastria immutabilis*) on Guadalupe Island was evaluated during the breeding seasons of 2015–2018 by measuring and comparing 10 morphological attributes: cranial length, bill length, nostril length, cranial width, bill height, bill width, tarsus length, closed wing length, opened wing length, and wingspan length in reproductive adults (*n* = 135). Males were larger than females across all traits (Student’s *t*-test, *p* < 0.05, *p* < 0.05). We created a logistic model using stepwise regression to predict sex based on morphological variables. This model indicated four significant morphological predictor variables (*z* < 0.05) and was able to successfully predict the sex of *P. immutabilis* individuals in more than 90% of the cases. Based on these predictor variables, a web app was developed to determine the sex of the Laysan albatross in the field, providing a non-invasive method for rapid data collection that reduces costs and handling times while improving conservation efforts. We tracked Laysan albatross (*n* = 36) during breeding seasons and found no significant differences between females and males for either trip length (GLMM, F = 0.017, DF = 1, 1, *p* = 0.917 > 0.05) or maximum trip distance (GLMM, F = 0.374, DF = 1, 1, *p* = 0.651 > 0.05). Our results suggest that both sexes show a strong preference to travel to highly productive coastal waters northeast of the breeding colony that are influenced by the California Current. The present research will serve to establish a baseline to protect this species on Guadalupe Island and highlights the importance of understanding sexual dimorphism in at-risk seabird species.

## 1. Introduction

Sexual dimorphism in vertebrates is a condition in which the sexes (male and female) of the same species exhibit differences in size, color, markings, characteristics, or even behavior [1,2,3,4]. These differences may be subtle or extreme and are subject to sexual selection. Sexual dimorphism in bird species can be observed in conformation, plumage coloration, or body size, with males, typically being larger and more colorful while females bear the responsibility of choosing the fittest male as a reproductive partner [5,6,7,8,9]. This is important in polygynous mating systems, as females tend to select larger males [10,11] that only contribute genetic material during copulation and no subsequent parental care [12,13,14,15,16]. However, the existence of sexual dimorphism may appear less evident in monogamous species such as the Laysan albatross (*Phoebastria immutabilis*) [17,18], a species that is difficult to sex from field observations and exhibits bi-parental care, which likely contributes to the lack of sexual dimorphism observed [19].

Although the Laysan albatross mostly in the Central Pacific (Northwestern Hawaiian Islands), this species colonized new sites in the eastern Pacific in 1983, specifically, Guadalupe Island, Alijos Island, and the Revillagigedo Archipelago (the islands of Roca Partida, San Benedicto, and Clarión) [20,21]. This expansion of the Laysan albatross breeding range resulted in high oceanic spatial segregation, reflected in differences between the two populations based on foraging habitat, behavior, and reproductive success [22]. Laysan albatross from the Hawaiian colonies forage in cold, subarctic waters and in the North Pacific Transition Zone [23], while albatross from the Eastern Pacific colonies forage in the California Current System [22]. Although spatial segregation has been documented in other albatross species, their foraging habitats have been poorly defined [24,25]. For this reason, it is important to document sexual segregation in species with subtle dimorphism in marine environments, where it is often difficult to identify the sexes based on morphological traits. Furthermore, the recognition of sexual segregation, which differs between potential feeding grounds in at-risk species, can contribute to the accurate evaluation of distribution areas and improve conservation efforts.

Sexual dimorphism has not been reported for Laysan albatross from Guadalupe Island. Species of the order Procellariiformes (albatrosses and petrels) have been shown to exhibit varying degrees of sexual dimorphism [26,27]. Males of the wandering albatross (*Diomedea exulans*), the black-browed albatross (*Thalassarche melanophris*), and the grey-headed albatross (*Thalassarche chrysostoma*) are bigger than females and exhibit sexual segregation through the use of distinct foraging zones [28,29]. Differences in certain morphological traits between the sexes of a given species may be of particular ecological importance (e.g., a greater wingspan permits the exploration of distant areas [28]) and leads to differential niche utilization by males and females [30]. Therefore, at-sea seabird distributions may be influenced by factors such as morphology, sex- or age-based competition, or environmental conditions, reflected in the spatial and temporal segregation of individuals within populations or between different species [31].

The Laysan albatross, like many other seabird species, is considered a sentinel of environmental change [32,33]. The at-sea distribution of this seabird is influenced by factors like ocean circulation, wind patterns, sea surface temperatures, habitat quality, and prey availability [28,34,35,36,37,38,39,40,41]. More specifically, the Laysan (*P. immutabilis*) and black-footed (*P. nigripes*) albatrosses from the Hawaiian Islands exhibit differences in the time spent foraging, distances travelled, and habitat utilization during the distinct reproductive stages that are: (1) the incubation period (parents alternate between fasting while incubating the egg and foraging at sea), (2) the brooding period (breeding pairs alternate between fasting at the nest and foraging at sea and provisioning the chick), and (3) the chick-rearing stage (breeding pairs forage independently at sea and return to the nest periodically to quickly provision the chick) [38]. The time spent foraging, the distances travelled, and uses of potential feeding grounds are constrained by the energy requirements of the different reproductive stages [23,24,42,43]. When comparing the different reproductive stages, it can be seen that individuals travel further during the incubation and chick-rearing stages compared to the brooding period [38].

Molecular, vocalization, and behavioral techniques have been developed to sex birds but they are costly, time-consuming, and require in-depth training. As such, a low-cost sex identification technique that reduces handling time in the field is needed. To address this challenge, we propose a sex prediction model for the Laysan albatross based on morphometric measurements and present a corresponding progressive web application that predicts sex using a minimal number of input variables with the potential for use in remote areas.

Understanding sexual dimorphism in the Laysan albatross on Guadalupe Island as well as addressing the complexity involved in evaluating the morphological, physiological, and behavioral traits of this species is essential for future evaluations of the biology and population dynamics of this at-risk seabird [44,45]. Therefore, the objectives of this study are as follows: (1) to evaluate the existence of sexual dimorphism in Laysan albatross on Guadalupe Island, (2) to determine which morphological traits present the greatest degree of differentiation between males and females, (3) to accurately predict the sex of Laysan albatross individuals with a minimal number of morphological input variables, and (4) to determine if there are differences in the distances travelled between males and females during the breeding season.

## 2. Materials and Methods

### 2.1. Data Collection

**Figure 1:**
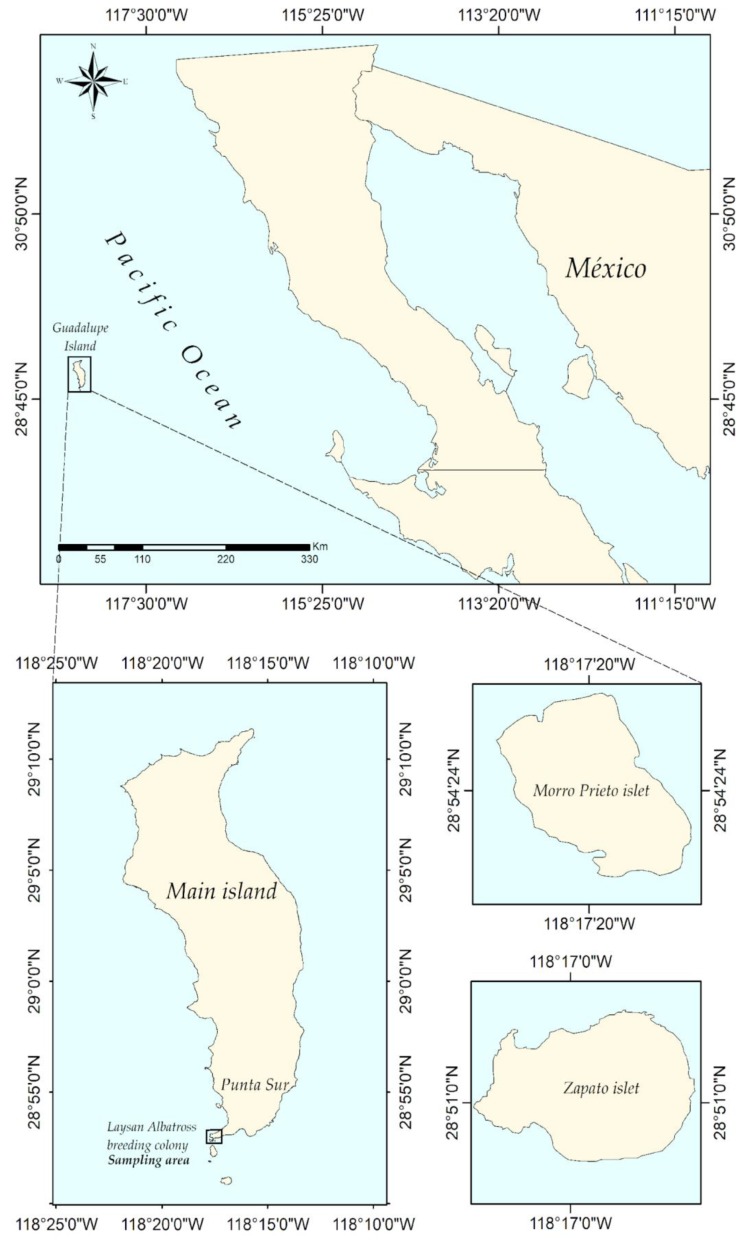
Top panel: Location of Guadalupe Island with respect to the Baja California Peninsula, Mexico. Bottom panels: Close-up of the locations of the Morro Prieto and Zapato islets with respect to the main island.

The research was conducted on Guadalupe Island (GI; 29°04′N, 118°17′W) under permits granted by the following Federal Government agencies: Secretaría de Gobernación, Secretaría de Medio Ambiente y Recursos Naturales, and Comisión Nacional de Áreas Naturales Protegidas. In addition, research activities were developed under permits granted by the Secretaría de Medio Ambiente y Recursos Naturales to collect biological material from wild species of flora and fauna. No albatross were harmed during this experiment. GPS tagging was carried out when the albatross was captured for blood sample collection and morphometric measurement. The GPS monitoring period took place during the breeding season, which lasts from November to June [20,46], and included the incubation, brooding, and chick-rearing stages. Albatross on GI are distributed between three different colonies: the Zapato islet, the Morro Prieto islet, and the main island, which are located 70–210 m above sea level [20,40] (Figure 1).

Guadalupe Island (29°04′N, 118°17′W) is of particular importance for Laysan albatross because the most successful breeding colony in the eastern Pacific is found on the island [22,40]. Guadalupe Island is considered an Important Bird and Biodiversity Area (IBA) for conservation given that it serves as a refuge for more than 133 bird species, 26 of which are categorized as at risk according to the Mexican government (NOM-059-SEMARNAT-2010). The Guadalupe Island breeding colony of Laysan albatross has been monitored since 2003 [22]. This population is in continuous growth and has consolidated to become the most important population of Laysan albatross in the eastern Pacific [20,22]. The conservation and restoration of the Guadalupe Island population is the result of actions to control and eradicate invasive alien species that have been carried out by Grupo de Ecología y Conservación de Islas, A.C., during the last 15 years [20]. In general, Laysan albatross are long-lived [47] with high apparent survival between 0.93 and 0.99 [48]. Only one chick per pair is produced every season [17,49]. Currently, more than 1200 breeding pairs of Laysan albatross nest on Guadalupe Island and its islets [50]. Laysan albatross colonies were monitored throughout the reproductive season from 2014 to 2018. Individuals were selected based on their reproductive success from previous seasons and the degree of accessibility to their nests to facilitate monitoring during the breeding season.

We collected blood samples from 135 Laysan albatross over four consecutive years (*n* = 30 for 2015, *n* = 66 for 2016, *n* = 9 for 2017, and *n* = 30 for 2018). Tarsus prick samples (~30 μL) were obtained by means of glass capillary collection with a single-use lancing device and spotted directly onto FTA^®^ (Flinders Technology Associates^®^, Whatman, Inc., New Jersey, USA) cellulose filter paper [51,52]. Blood spots were allowed to dry for 2 h under ambient conditions and were then stored for shipment in desiccated bags.

We determined the sex of 135 Laysan albatross using genetic techniques. Total DNA was extracted from dried blood spots on FTA cards using a salt extraction protocol [53]. The extracted DNA was stored at −20 °C until the polymerase chain reaction (PCR) analysis was carried out using the primers 2550F (5′GTTACTGATTCGTCTACGAGA3′) and 2718R (5′ATTGAAATGATCCAGTGCTTG3′). All samples were run on 1.5% agarose gel and checked for a single (male) or double (female) bands [54]. Molecular analyses were conducted in the Molecular Ecology Laboratory of the Universidad Autónoma de Baja California in Ensenada, Baja California, Mexico.

**Figure 2:**
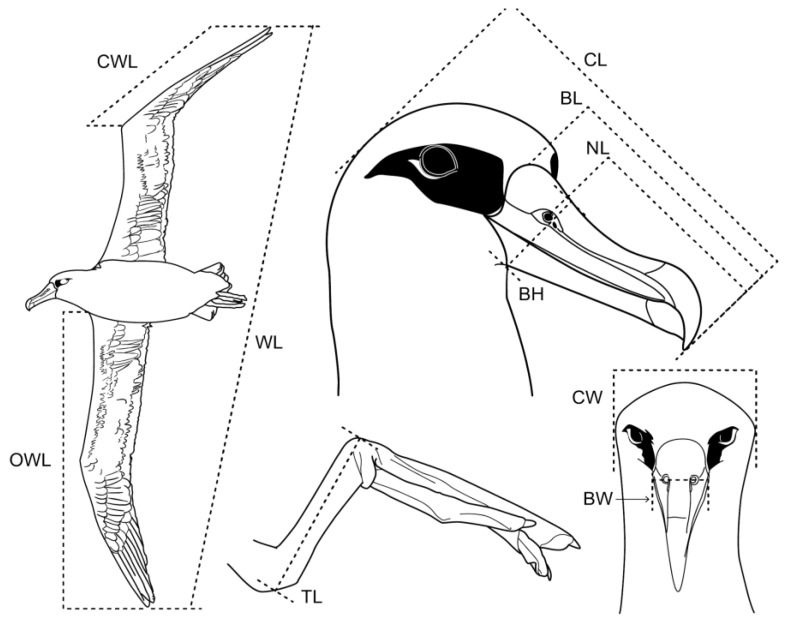
Diagram of the morphological measurements taken from reproductive Laysan albatross individuals.

We measured 10 morphological variables of Laysan albatross adults: cranial length (CL), bill length (BL), nostril length (NL), cranial width (CW), bill height (BH), bill width (BW), tarsus length (TL), closed wing length (CWL), opened wing length (OWL), and wingspan length (WL) using Vernier calipers with an accuracy of ±0.1 mm (Figure 2) [55,56]. All measurements were taken by the same person each year during the breeding seasons of 2015–2018. We also measured body mass with a Pesola digital pocket scale MS500 (precision: ±0.1 g), although this variable was not included in every analysis. Albatross were handled for short periods (5–8 min) to minimize stress.

We also used geographic positioning system (GPS) loggers (2014–2016: model GiSPy-4SB, Roma, Italy; 2017–2018: model i-gotu, Taipei, Taiwan) to track 36 Laysan albatrosses (*n* = 14 males, and *n* = 22 females) from 2014–2018. The weight of each GPS logger was 15–21 g. The GPS loggers were attached to the back feathers of albatross individuals by Tesa^®^ tape (No. 4651, Tesa AG, Hamburg, Germany) [24,57]. The GPS loggers were programmed to simultaneously record both the position and the instantaneous speed of the albatross every 20 min. The GPS loggers were able to record continuously for 12–15 days (model i-gotu) or 60–70 days (model GiSPy4SB) with these settings. The GPS monitoring period took place during the breeding season, including the incubation, brooding, and chick-rearing stages. The GPS were installed on the birds from 20 January to 20 April 2014, from 13 December to 24 March 2015, from 25 February to 17 April 2016, from 15 February to 16 March 2017, and from 18 January to 10 February 2018.

### 2.2. Tracking Data Processing

To define each trip, we filtered data to remove resident trips (trips with distances below the set threshold of 60 km) around the colony and eliminated short-period distant trips [58]. No interpolation was carried out to smooth the data. For each trip, we calculated the maximum trip distance (the furthest distance recorded from the origin point on the Guadalupe Island colony, 28.85°N, 118.2833°W) and trip length (the total distance travelled, i.e., the sum of the distance between contiguous points). We filtered out unreliable data points, which were attributed to tag failure and we only included those data points with longitudes greater than 0°W. To avoid bias that could arise from the potential contribution of multiple trips from each individually tracked bird, we treated individual as a random effect in statistical analyses.

To define foraging areas, we used kernel density. The 50% contour of trajectory kernel density has been associated with seabird foraging areas [23,59,60]. We calculated trajectory kernel density using the KernelDensity function from sklearn.neighbors package with a bandwidth of 0.005 and a cell size of 100 × 100 using haversine metrics.

### 2.3. Statistical Analysis

To evaluate sexual dimorphism in Laysan albatross from Guadalupe Island, we compared all morphological measurements between the sexes using the Student’s *t*-test for independent samples. All morphological variables were normally distributed (*p* > 0.05, in all Shapiro-Wilk’s one-sample tests). We also verified the homoscedasticity of the data set (homogeneity of variance between and within samples) using Levene’s test before performing each analysis. Body mass was the only variable that did not satisfy the homoscedasticity assumption (*p* = 0.008). As such, we eliminated this variable in subsequent analyses. We report the results in box plots for each measurement where we summarize the data displaying the minimum value, second quartile, median value, third quartile, and maximum value. We also obtained the mean values ($\bar{x}$), standard deviations (SD), and coefficients of variation (CV). In order to characterize the overall variability of morphological variables, we carried out a principal component analysis (PCA) after transforming the data to ensure comparable scales with the equation:

(1) $$x=\frac{x_{i}-µ}{\sigma }$$

All statistical analyses were performed using the R studio package 3.0.1 [61].

A logistic model was used to estimate the probability of an individual albatross being male or female based on the values of eight morphological characteristics (we removed nostril length and closed wing length because of their similarity to bill length, and opened wing length, respectively). The logistic model was created using stepwise regression, which is a semi-automated process of building a model by successively adding and removing variables, combining forward and backward selection techniques [62]. Stepwise regression begins with a model that contains no predictors, followed by the successive addition and removal of single predictors to the model in order to find a model with the lowest Akaike Information Criterion. To create the model, we randomly selected 108 (80%) observations as a training sample and used the remaining 27 (20%) observations as a validation sample. We use the training sample to fit the model. The fitted model was applied to the validation sample, and the performance of the model was evaluated.

**Figure 3:**
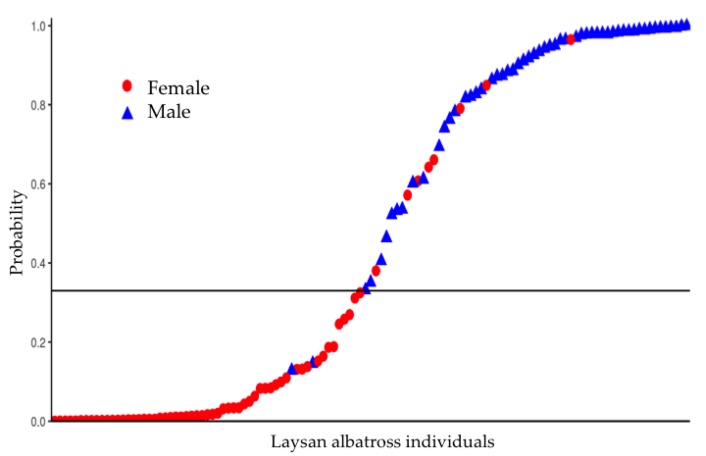
Sex prediction logistic model based on morphometric data (females shown as red circles and males shown as blue triangles). The black line is the threshold defined by the receiver operating characteristic (ROC) curve to decide which model output values will be considered male and which female. The sex of albatross individuals was successfully predicted in the >90% of the cases.

We used a receiver operating characteristic (ROC) curve to calculate the threshold with which to interpret the output of the model. Individuals whose morphological attribute values fell above the threshold were considered male and those that fell below were considered female (Figure 3).

The model followed these equations:

(2) $$\frac{1}{1+e^{-z}}$$

where *z* is

(3) $$z=\sum_{i=0}^{n}\beta_{i}\psi_{i}$$

(4) $$\psi_{i}=N_{i}\psi_{i}$$

(5) $$N_{i}\left(x_{i}\right)=\frac{x_{i}}{\max\left(x\right)-\min\left(x\right)}$$

where $\beta_{i}$ is a coefficient/parameter, $N_{i}$ is the normalizing function, and $x_{i}$ is the morphological attribute.

To test for differences in trip length and maximum trip distance between females and males, we used a GLMM (General Linear Mixing Model), where year (inter-annual variability) and individual was treated as a random factor.

## 3. Results

### 3.1. Genetic Sex Identification

Of the 135 albatrosses that were sexed via genetic analyses, 74 were females and 61 were males.

### 3.2. Morphological Dimorphism

**Figure 4:**
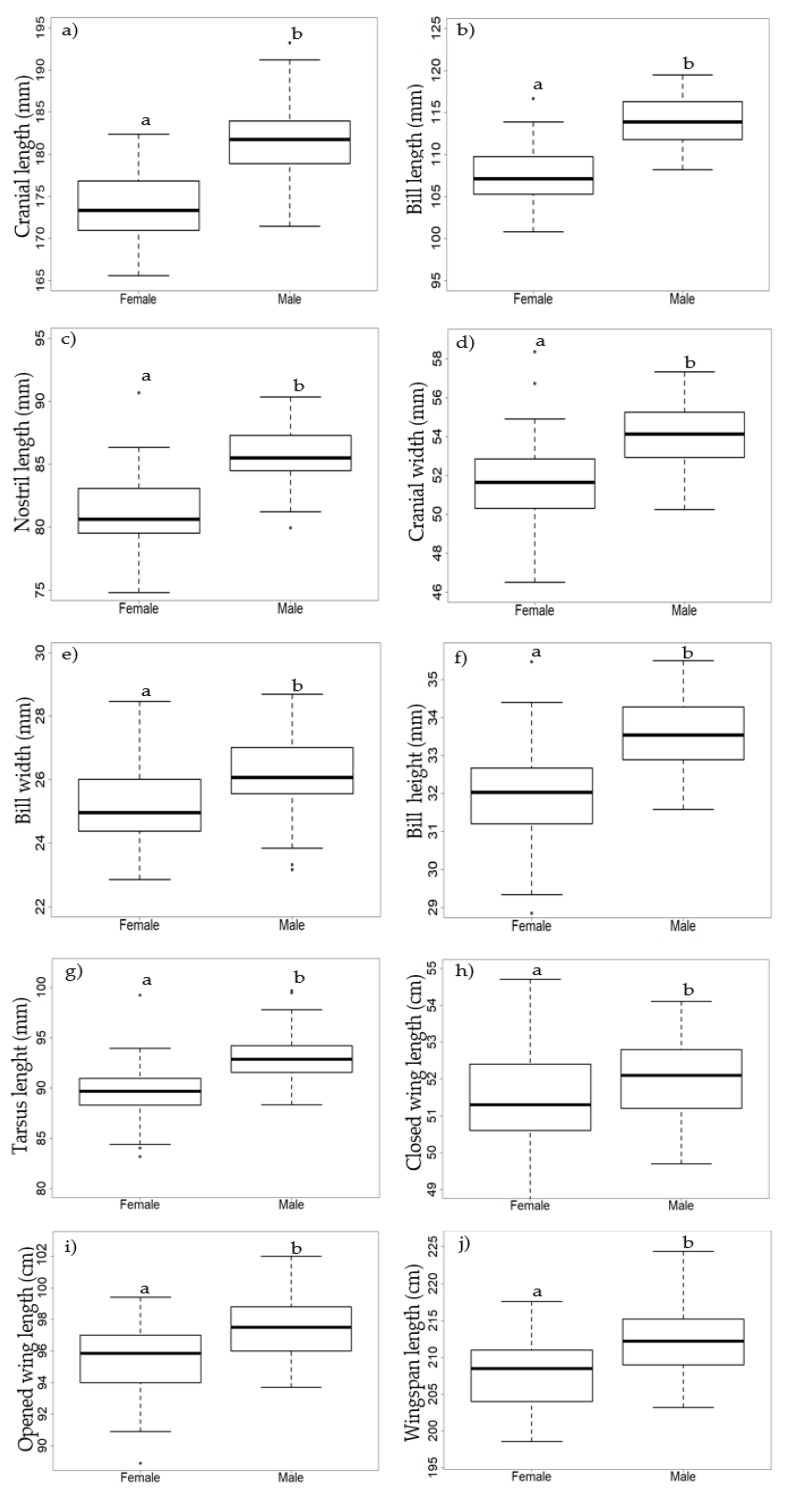
Box plots of the morphological variables for females (*n* = 74) and males (*n* = 61) of Laysan albatrosses collected from 2015–2018. The bold line represents the median, the bottom of the box is the first quartile, the third quartile separates 75% of the data, the points above are the limits of the whiskers (shown as dashed lines), and the extreme values (atypical data) or outliers are shown as open circles. Different letters (**a**/**b**) are used to indicate significant differences.

Males were significantly larger than females across all morphological traits (Student’s *t*-test, *p* < 0.05). The morphological variables that presented the greatest differences were the cranial length (Figure 4a), bill length (Figure 4b), nostril length (Figure 4c), bill height (Figure 4f), and tarsus length (Figure 4g). Females presented a high coefficient of variation for most morphological traits, in contrast with males that showed greater homogeneity of variance. Although body mass was not considered in sexual dimorphism analyses, males were significantly heavier than females despite the presence of high intra-variation (Table 1).

The PCA characterized the variability of all morphological variables in two principal components, which explained 68% of the total variance. The first component explained a 54% of the variance of the morphological data and the second axis described 14% of the total variance. The first principal component (PC1) was mainly related to cranial length, bill length, and nostril length, associated with the size of the individuals. PC1 was normally distributed, with males showing higher values (*p* < 0.01). The second principal component (PC2) was mainly related to closed wing length, opened wing length, and wingspan length, which are traits generally associated with flight. PC2 was normally distributed. In all measurements, significant sex differences were found. Subsequent components showed variances of less than 10% (Table 2).

### 3.3. Sex Prediction Model

The stepwise regression identified four variables as the best predictors: tarsus length, cranial width, bill height, and bill length (Table 3). The first two columns in Table 3 contain the name of the coefficient name and the estimated value. Standard errors of the estimated coefficients are presented in the third column. The fourth column displays the *z*-value, the ratio of the estimated coefficients to their estimated standard errors. If the *z*-value is large in magnitude, the corresponding true regression coefficient is not zero. Since the sample size is small, we repeated the fitting and validation process 2000 times. In each iteration, we randomly selected 80% of the observations. We selected a set of models with better performance for the validation and assessed the set with the complete dataset. The threshold calculated by the ROC curve was 0.33. The model correctly predicted the sex of albatross individuals in >90% of the cases.

**Figure 5:**
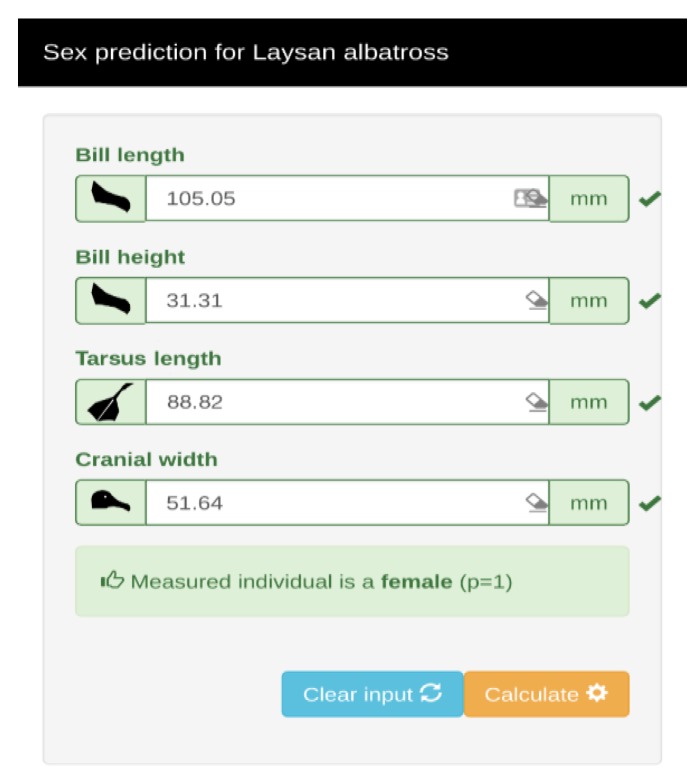
Visual example of the sex prediction app for Laysan albatross. The application developed to predict the sex of the Laysan albatross will be available to any user. The application will be available as of August 2019 and will found on the site: app.islas.org.mx/laysan-albatross-sexual-dimorphism.

We developed a software application (app) to predict the sex of Laysan albatross adult individuals (Figure 5). This app asks for four morphological input measurements: (1) bill length, (2) bill height, (3), tarsus length, and (4) cranial width, which were the best morphological predictors of our sex predictor model. This app has the potential for use in any remote place.

### 3.4. Laysan Albatross Foraging Trips

The mean trip length was greater for males than females, in contrast to the mean maximum trip distance, which was greater for females (Table 4). We used a GLMM to test this observation statistically; however, there were no significant differences between females and males for either trip length (GLMM, F = 0.017, DF = 1, 1, *p* = 0.917 > 0.05) or maximum trip distance (GLMM, F = 0.374, DF = 1, 1, *p* = 0.651 > 0.05) considering year and individual as a random effect.

**Figure 6:**
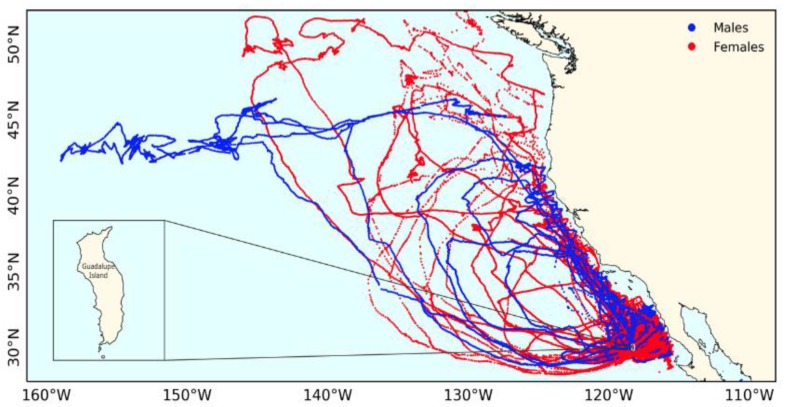
Trips made by Laysan albatross (*n* = 36) males (blue lines) and females (red dashed lines) during breeding seasons of 2014–2018 on Guadalupe Island obtained with GPS.

The distribution patterns of Laysan albatross foraging trips during the breeding seasons of 2014–2018 were concentrated along the near shore zone of the eastern Pacific (Figure 6), but no strong evidence of spatial sexual segregation was found.

**Figure 7:**
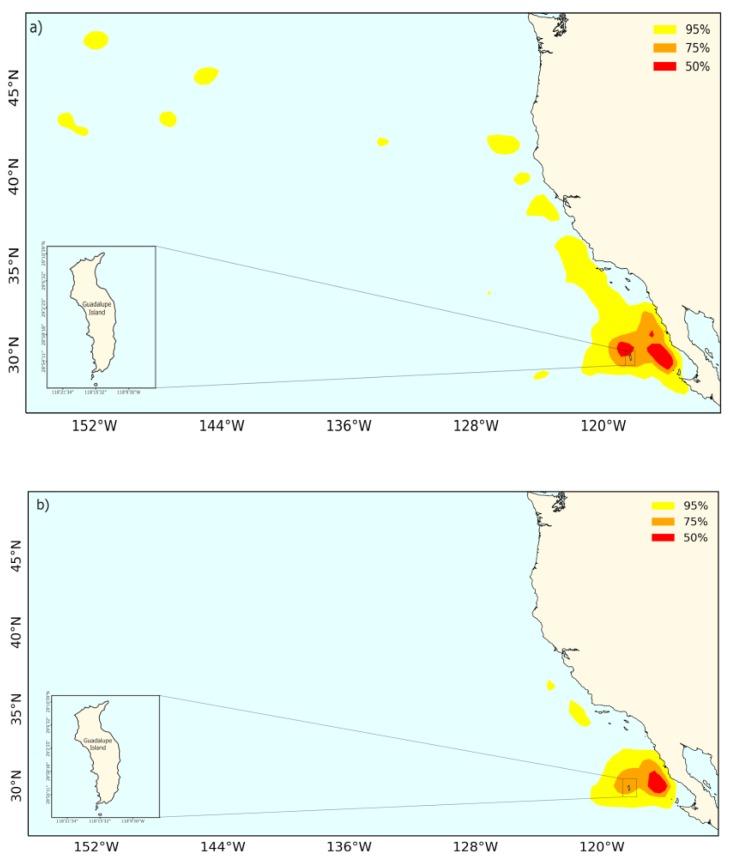
Contour maps of the trajectory kernel density of Laysan albatross for (**a**) males and (**b**) females during breeding seasons of 2014–2018 on Guadalupe Island. Red, orange, and yellow colors represent 50%, 75%, and 95% kernel densities, respectively.

The contour map kernel (50%) indicated that there was no evidence to suggest that males exploit different foraging areas compared with females during the breeding season on Guadalupe Island (Figure 7).

## 4. Discussion

### 4.1. Sexual Dimorphism

Sexual selection can play a major role in the evolution of morphological traits in birds and morphological differences between the sexes are often related to physiology or external pressures [1,63,64]. We found sexually dimorphic traits in the Laysan albatrosses on Guadalupe Island that were most evident for four attributes: tarsus length, cranial width, bill height, and bill length. However, the differences are not as evident as in other species like the wandering albatross (*Diomedea exulans*) or northern giant petrel (*Macronectes halli*) [56,64]. Tickell et al. [65] evaluated sexual dimorphism in the wandering albatross on Bird Island, South Georgia, UK, and found significant differences between the sexes in weight, wingspan, culmen, depth of bill, tarsus, and mid-toe length, with males generally being larger than females. Our results indicated differences in almost all of the same traits reported by other authors for similar species. Bill length and wingspan have been reported to be good measurements to differentiate sex [66,67]. The morphological differences found between Laysan albatross males and females in our study are less marked than those found for other albatross species, such as the black-brown albatross [68,69,70]. For example, an average 7.2% difference in mass between males and females was reported for the Campbell albatross and a 20.2% difference was reported for the black-browed albatross [28]. In our study, we found a difference of 14.7% for the same trait, indicating that a medium degree of sexual dimorphism was present in the Laysan albatross compared with other albatross species.

The Laysan albatross is socially monogamous, long-lived, and exhibits bi-parent care [19]. However, Laysan albatrosses have been documented in long-term same-sex couples (female–female) that pair for the care and feeding of young [52]. This cooperation between individuals of the same sex could result in a skewed sex ratio in the reproductive population, although further research of this important topic is needed. For example, 31% of Laysan albatross breeding pairs on Oahu, Hawaii, were female–female, and the overall sex ratio was 59% female due to sex-biased immigration. The sex ratio of a population can change social structures and cause cooperative behavior to arise in monogamous species, which emphasizes the importance of sexing monomorphic species and detecting the degree of dimorphism present [52]. To this end, investigating the prevalence of cooperative breeding using morphometric inference, such as in the present study, could greatly contribute to understanding Laysan albatross population dynamics on Guadalupe Island.

### 4.2. Sexual Segregation

Sexually dimorphic seabird species exhibit spatial segregation and at-sea distributions constrained by their individual attributes (e.g., morphology, color, and size), environmental factors (e.g., prey availability, species-level interactions, physiological limits, dispersal patterns, and migratory behavior) [28,38], and by breeding stage, which depend on the energy requirements of the species [28,31,71,72,73].

Although Laysan albatross showed some degree of sexual dimorphism, no significant differences were found in the distances traveled between males and females. This result contrasts with a previous study by González-Solis [56] that reported differences in foraging efforts (i.e., flight speed, distances covered, and the duration of foraging trips) between females and males of the giant petrel (*Macronectes halli*) in South Georgia. This contrast in results may be due to the differences in sexual dimorphism observed between these two seabird species given that *M. halli* is the most sexually dimorphic of all seabird species.

Laysan albatrosses from the Northwestern Hawaiian Islands have to make long foraging trips around their breeding colonies due to reduced prey abundance [19,74,75,76,77]. In contrast, the feeding trips of Laysan albatrosses from Guadalupe Island are directed towards highly productive marine zones northeast of their reproductive colony, which are the result of coastal upwelling and the influence of the California current. The fortuitous proximity of a highly productive foraging zone to the breeding colony favors Laysan albatross population growth of on Guadalupe Island [20] and eliminates the need for individuals to travel further in search of feeding grounds, regardless of sex-based morphological differences in this species that may limit or facilitate the ability to travel. A similar relationship has been found between the proximity of productive foraging zones and the breeding and nesting areas of the waved albatross (*Phoebastria irrorata*); specifically, equatorially nesting albatross had greater access to the highly productive waters of the Humboldt Current, to localized upwelling, and to equatorial fronts during brooding [38,78].

Sexual segregation of foraging habitats is relatively common in the wild [31,79] and has been well documented for albatross [23,24,25] and other bird species like waders, passerines, and raptors, with males having been shown to cover considerably greater distances than females [79]. This tendency may be due to males being generally larger and therefore able to travel greater distances, or to factors like flight proficiency, which has been associated with larger wingspans and the ability to fly farther [80]. In our study, the males appeared to have slightly more potential feeding grounds than females. This tendency towards sexual segregation may vary along temporal and spatial scales; however, we did not find any strong evidence of sexual segregation in Laysan albatross from Guadalupe Island. The sexual dimorphism present in Laysan albatross from Guadalupe Island, in which males were generally larger than females, was not associated with greater distances travelled by males.

Sexual segregation in foraging areas is also linked to the specialization of reproductive functions, which has been observed in the black-browed albatross and the gray-headed albatross [28]. Furthermore, studies carried out on the Northwestern Hawaiian Islands have shown interspecific segregation of core foraging areas between Laysan and black-footed albatross during incubation and chick-rearing stages but not during the brooding stage [38], as long trips were needed to meet nutritional demands, particularly during the incubation and chick-rearing stages [23,24,43]. In particular, Conners [42] found differences in the distances traveled during foraging trips in the brooding period, with females flying farther away from the breeding colony. These results encourage us to carry out a more detailed investigation regarding the distances traveled by Laysan albatross on Guadalupe Island during the different reproductive stages.

Food resources can be a fundamental factor that influences population growth in wild species [81]. When competitive interactions are severe, they are assumed to be related to population density [82]. However, the California Current System appears to provide rich and highly productive waters in which Laysan albatross can feed under conditions that are apparently free from intraspecific competition. Furthermore, the Laysan albatross population from Guadalupe Island is comprised of 1300 reproductive pairs [20,50], which makes it relatively small compared to the Hawaiian colony. As indicated by Ashmole [83], individuals from larger colonies will have to travel further to find food for their chicks than those from smaller colonies given that prey abundance in waters surrounding lager colonies is likely reduced [84]. In addition, *P. immutabilis* shares the same breeding season as the Laysan albatross on Guadalupe Island; however, interspecific competition for food resources has not developed. Nonetheless, to determine which factors regulate density-dependent growth population in the Laysan albatross on Guadalupe Island, it is essential to continue monitoring this species.

Our results indicate that the temporal distribution and potential feeding grounds of the Laysan albatross could be site specific, which may result in the development of diverse feeding and reproductive strategies. Although this study provides a baseline that represents the first attempt to understand the roles played by sexual dimorphism and feeding trips of the Laysan albatross from Guadalupe Island, we know that many other environmental, ecological, and biological factors may constrain the foraging potential and distribution areas of this species. More detailed analyses are required in the future that specifically evaluate the potential impact of fisheries on albatross reproductive and foraging behaviors in order to facilitate the protection of the Laysan albatross on Guadalupe Island, as this colony is the most successful and important Laysan albatross colony in the eastern North Pacific [20,22]. Furthermore, it is quite possible that sexual dimorphism in Laysan albatross from Guadalupe Island has arisen from sexual selection, where the presence of larger, dominant males leads to the division of roles or niche specialization, especially during the breeding season. Future research regarding the behavior and habitat use of the Laysan albatross is important for the protection and conservation of this species.

## 5. Conclusions

Laysan albatross reproductive males on Guadalupe Island were significantly larger than reproductive females. Tarsus length, cranial width, bill length, and bill height were the morphological variables that were best able to demonstrate sex-based differences in the reproductive population of Laysan albatross from Guadalupe Island.

A sex predictor model and a corresponding web app were developed to identify the sex of Laysan albatross on Guadalupe Island. The web app has proved to be an efficient tool that allows for rapid data collection and processing while eliminating the use of invasive sampling techniques. In addition, the web app will reduce the financial costs involved in future research endeavors as well as the handling time involved in sampling Laysan albatross individuals. The web app allows for the real-time sexing of an individual, which provides multiple advantages for monitoring this species in the field. In addition, the app may be used in remote areas and by anyone without the need for specialized training. Lastly, the low-cost web app increases the opportunities to expand our knowledge of this species and will facilitate future conservation efforts.

We found no significant differences between the distances travelled by Laysan albatross females versus males during the breeding season. Our results suggest that both sexes show a tendency to travel northeast of their breeding colony to productive, coastal waters on the continental shelf of the Baja California peninsula that are influenced by the California Current.

Information regarding the distribution, abundance, behavior, seasonality, and threats to bird colonies is necessary to identify Important Bird and Biodiversity Areas (IBA) that comprise Laysan albatross habitat. We believe the present research could provide a baseline for future studies that aim to identify areas through the use of GPS tracks that must be protected for the conservation of the Laysan albatross in the northeastern Mexican Pacific.

## Figures and Tables

**Table 1:** Comparisons between male (*n* = 61) and female (*n* = 74) Laysan albatrosses on Guadalupe Island from 2014–2018 for all morphological variables. The mean ($\bar{x}$), standard deviation (SD), and coefficient of variation (CV) are presented.

Variable	Male			Female			*t*-Test	*p*-Value
	$\bar{x}$	SD	CV	$\bar{x}$	SD	CV		
Cranial length (mm)	181.80	4.10	2.26	173.61	3.92	2.26	−11.84	<0.001
Bill length (mm)	113.88	2.64	2.32	107.56	2.93	2.72	−13.05	<0.001
Nostril length (mm)	85.66	2.14	2.50	81.19	2.69	3.32	−10.52	<0.001
Cranial width (mm)	54.15	1.56	2.89	51.67	2.00	3.86	−7.91	<0.001
Bill height (mm)	33.57	0.91	2.71	32.01	1.27	3.96	−8.08	<0.001
Bill width (mm)	26.20	1.19	4.55	25.12	1.34	5.32	−4.90	<0.001
Tarsus length (mm)	93.14	2.29	2.46	89.61	2.52	2.81	−8.44	<0.001
Closed wing length (cm)	52.03	1.05	2.01	51.40	1.26	2.45	−3.10	0.002
Opened wing length (cm)	97.46	2.07	2.12	95.58	2.16	2.26	−5.14	<0.001
Wingspan length (cm)	212.66	4.14	1.95	207.63	4.72	2.28	−6.50	<0.001
Body mass (kg)	2.86	0.34	11.79	2.44	0.23	9.54	−8.55	<0.001

**Table 2:** Principal components analysis with the 10 morphological traits of Laysan albatross on Guadalupe Island collected from 2014–2018.

Variable	PC1	PC2
Cranial length	−0.38	0.14
Bill length	−0.37	0.23
Nostril length	−0.37	0.23
Cranial width	−0.27	0.25
Bill height	−0.29	0.28
Bill width	−0.27	0.21
Tarsus length	−0.33	0.13
Closed wing length	−0.23	−0.60
Opened wing length	−0.30	−0.46
Wingspan length	−0.34	−0.33
Standard deviation	2.32	1.17
Proportion of Variance	0.54	0.14
Cumulative Proportion	0.54	0.67

**Table 3:** Predictor variables.

Variable	Estimate	Std. Error	*z* Value	Pr (>|z|)
(Intercept)	−12.466	2.765	−4.508	0
Bill length	8.661	2.624	3.301	0.001
Bill height	5.911	2.662	2.221	0.026
Cranial width	3.612	1.851	1.952	0.051
Tarsus length	4.234	2.254	1.878	0.06

**Table 4:** Foraging trip characteristics of Laysan albatross on Guadalupe Island.

Sex	*n*	No. of Trips	Trip Length (km)		Maximum Trips Distance (km)	
			$\bar{x}$	SD	$\bar{x}$	SD
Males	16	96	2270.5	3849.60	401.00	415.80
Females	20	148	1874.7	2808.20	420.90	491.50
